# Bacterial Extracellular Polysaccharides Involved in Biofilm Formation

**DOI:** 10.3390/molecules14072535

**Published:** 2009-07-13

**Authors:** Barbara Vu, Miao Chen, Russell J. Crawford, Elena P. Ivanova

**Affiliations:** 1 Faculty of Life and Social Sciences Swinburne University of Technology, PO Box 218, Hawthorn, Victoria 3122, Australia; 2 CSIRO Minerals, Bayview Avenue, Clayton, Victoria 3168, Australia

**Keywords:** extracellular polymeric substances, biofilms, bioremediation, acidithiobacillus ferrooxidans

## Abstract

Extracellular polymeric substances (EPS) produced by microorganisms are a complex mixture of biopolymers primarily consisting of polysaccharides, as well as proteins, nucleic acids, lipids and humic substances. EPS make up the intercellular space of microbial aggregates and form the structure and architecture of the biofilm matrix. The key functions of EPS comprise the mediation of the initial attachment of cells to different substrata and protection against environmental stress and dehydration. The aim of this review is to present a summary of the current status of the research into the role of EPS in bacterial attachment followed by biofilm formation. The latter has a profound impact on an array of biomedical, biotechnology and industrial fields including pharmaceutical and surgical applications, food engineering, bioremediation and biohydrometallurgy. The diverse structural variations of EPS produced by bacteria of different taxonomic lineages, together with examples of biotechnological applications, are discussed. Finally, a range of novel techniques that can be used in studies involving biofilm-specific polysaccharides is discussed.

## 1. Introduction

Microorganisms are traditionally studied, characterized and identified as planktonic, single cells [1,2,3]. However, detailed studies of sessile communities in different environments have lead to the conclusion that planktonic microbial growth rarely exists in nature. The investigation of microbial aggregates on tooth surfaces by Antonie van Leeuwenhoek resulted in the identification of microbial biofilms [1,4,5]. Heukelekian and Heller found that for marine microbes, growth and activity were enhanced by the presence of a surface onto which they could adhere [6]. During a study of natural marine bacteria populations, Zobell also discovered that there were many more microbes found attached to solid surfaces than were found in the surrounding medium [7]. It has become evident that bacterial function and growth within a population is a fundamental aspect of bacterial survival and a typical life style of microorganisms [8].

The term ‘biofilm’ was coined and described in 1978 [9]. Since then, it has been well documented that biofilm-associated microbes differ from their planktonic relatives in terms of the genes that are transcribed [1]. Bacteria can develop biofilms on a number of different surfaces, such as natural aquatic and soil environments, living tissues, medical devices or industrial or potable water piping systems [1,10]. Clusters of different microbial populations are found in almost all moist environments where nutrient flow is available and surface attachment is possible [11]. Biofilms have been found to protect the microbial community from environmental stresses [10,11,12]. This is why the formation of biofilms in natural and industrial environments allow bacteria to develop resistance to bacteriophage, amoebae, chemically diverse biocides, host immune responses and antibiotics [5]. These important characteristics have resulted in biofilm science and biofilm engineering emerging as intensively developing areas of research [13].

## 2. Microbial extracellular polysaccharides as an integral part of bacterial biofilms

A biofilm can be defined as an aggregation of bacteria, algae, fungi and protozoa enclosed in a matrix consisting of a mixture of polymeric compounds, primarily polysaccharides, generally referred to as extracellular polymeric substance (EPS). Over 99% of microorganisms on Earth live within these biopolymers. The formation of biofilms is a prerequisite for the existence of all microbial aggregates [14,15] as an essential step in the survival of bacterial populations [16]. The proportion of EPS in biofilms can comprise between approximately 50-90% of the total organic matter [1,10]. In Gram-negative bacteria, some of the polysaccharides are neutral or polyanionic. The presence of uronic acids or ketal-linked pyruvates enhances their anionic properties, thus allowing the association of divalent cations such as calcium and magnesium to increase the binding force in a developed biofilm. In some gram-positive bacteria, the chemical composition of their EPS could be slightly different due to their primarily cationic nature [1,15]. Aside from polysaccharides, biofilms also consist of proteins, nucleic acids, lipids and humic substances. Often the composition and quantity of the EPS will vary depending on the type of microorganisms, age of the biofilms and the different environmental conditions under which the biofilms exist [17]. These include different levels of oxygen and nitrogen, extent of desiccation, temperature, pH, and availability of nutrients. Their existence in such a range of environments suggests that these microorganisms are able to respond to their environments and change their EPS and adhesion abilities, depending on the properties of the surface onto which they attach [12]. It has been reported that microbial colonization on solid surfaces can be affected by diverse range of parameters. For example, the degree of colonization of certain surfaces has been found to increase with surface roughness because the ‘valleys’ present can allow the microbes to reside in a protected area with reduced shear forces and the surface roughness provides a surface with increased surface area for bacterial attachment [1]. Also, microorganisms have been found to attach more rapidly to hydrophobic and non-polar surfaces than hydrophilic surfaces [1,10]. Cell surface hydrophobicity, the presence of fimbriae and flagella and the degree of EPS production are other main factors that have been shown to profoundly influence the rate and degree of attachment of microbial cells to different surfaces [1]. The three types of forces involved in this process are electrostatic interactions, hydrogen bonds and London dispersion forces [10,17]. These binding forces are likely to contribute to the overall stability of biofilm matrices [10]. Different components of EPS have also been found to influence the extent to which microorganisms can adhere to both hydrophilic and hydrophobic surfaces. It has also been shown that the formation of EPS leads to irreversible attachment with different environmental surfaces [1,16,18].

## 3. Role of EPS in the regulation of biofilm formation

The growth of a biofilm is the result of a complex process that involves the transport of organic and inorganic molecules and microbial cells to the surface, a subsequent adsorption to the surface and finally an irreversible attachment aided by the production of EPS [19]. Due to its complexity, the formation of biofilms is regulated at different stages via diverse mechanisms [20,21]. The most studied regulatory mechanism that has been found to control the production of EPS, biofilm formation and differentiation is quorum sensing (QS) regulation [1,20,21,22,23,24]. QS allows bacteria to maintain cell-cell communication and also regulate the expression of specific genes in response to changes in cell population density [23,25]. In general, the QS process involves the production, release and detection of chemical signalling molecules, thus allowing microbial cells to regulate gene expression in a cell-density-dependent manner [26]. At a given population density, the genes involved in biofilm differentiation and maturation are activated [1,21].

Two QS processes have been described for bacteria [21,27]. The autoinducer-1 (AI-1) type is mainly involved in intra-species communication and the AI-2 type is associated with inter-species interaction [28]. Gram-negative bacteria produce and release AI molecules, which are generally *N*-actyl homoserine lactone (AHL) molecules that serve as a function of controlling the cell-population density. Bacteria detect the accumulation of AHL signals. Above a certain threshold concentration, these signals are present in sufficient quantity to enable similar transcriptional effectors to activate silent genes. This alters their cell-density dependent gene expression and therefore their behaviour [20,22]. In Gram-positive bacteria, communication is carried out with modified oligopeptides generating the signals and membrane-bound sensor histidine kinases acting as receptors. Signalling is mediated by many phosphorylation steps, which control the activity of a response regulator. However, peptide signals are not diffusible across the membrane and therefore the signal release is mediated by oligopeptide exporters. Normally, signal release occurs concurrently with signal processing and modification [21,27].

One of the best studied QS processes is the AHL-mediated QS system first described for the bioluminescent marine bacterium *Aliivibrio fischeri* (formerly *Vibrio fischeri* [29]). This system is considered to be the ideal model for the QS paradigm in most gram-negative bacteria. There are two proteins, LuxI and LuxR, which control the expression of the luciferase operon required for light production in *A. fischeri*. LuxI is the autoinducer synthase that produces AHL inducers via S-adenosylmethionine (SAM) and LuxR is the cytoplasmic inducer receptor/DNA-binding transcriptional activator that requires AHL coinducers to initiate expression of the luciferase-coded function [21,22,27]. Once produced, AHL molecules diffuse in and out of the cell membrane and increase in concentration when the cell-population density increases. When the critical threshold concentration is reached, the AHL is bound by LuxR. The resulting LuxR-AHL complex activates the transcription of the luciferase operon, and also the expression of LuxI and other genes involved in different behavioural responses, creating a positive feedback loop, which results in the production of light [21,23,27].

## 4. Control of EPS production

QS is known as one of the regulatory pathways for EPS production and biofilm formation in bacteria [20,23,26,27,30]. Also, phosphate and polyphosphate metabolism has been associated with biofilm development and the QS regulatory pathway [28,31]. However, since the QS regulatory system and biofilm formation and maintenance mechanisms are diverse among different bacterial species, the role of QS in biofilm formation cannot be described in general terms [26]. For example, in *Pseudomonas aeruginosa*, QS is essential for adhesion, proper biofilm formation and virulence factors [21,32]. They have two QS systems, LasI/LasR and RhlI/RhlR, which work the same way as the LuxI/LuxR system in *A. fischeri* [27]. Mutant *P. aeruginosa* cells that did not produce any QS signals were found to be more densely populated with a thinner biofilm than the wild type. In addition, mutation of the LasI gene resulted in an abnormal and undifferentiated biofilm formation process [24]. In *E. coli*, cellular functions are controlled by the QS LsrR/LsrK system. Biofilm formation and architecture were found to be significantly altered in *lsrR* and *lsrK* mutants. There were differences observed in the cell fimbriae and matrix structure and in the thickness of the mutants compared to the wild type [33]. Lastly, a QS system, AfeI/AfeR, has recently been identified in *Acidithiobacillus ferrooxidans*, which is similar to the LuxI/LuxR proteins [20,23]. It was found that the amount of lipopolysaccharides present was increased in phosphate starved *A. ferrooxidans*. There was also an increase in the transcription of the *afeI* gene, and therefore AHL levels, when cells were cultured in a low-phosphate medium compared to that of a high-phosphate medium. Since the AHL communication system is present in *A. ferrooxidans*, this suggests that QS could regulate the formation of EPS and biofilms for attachment to solid substrates [20,28].

## 5. EPS implications in biotechnology

There is a great deal of interest in the EPS of microorganisms used in the food, pharmaceutical, biomedical, bioremediation and bioleaching fields due to their wide structural diversity and their physical, rheological and other unique properties [34,35]. An expanding area of biotechnology is the application of microorganisms in the remediation of environmental effluents produced by the mining and metallurgy industries [25,36,37]. Various strategies have been developed to eliminate contaminants from the environment and to reduce the amount of toxic waste entering the environment from these industries [11,38]. Over the past few decades, the use of microbes in remediation techniques has increased in popularity due to their efficiency and their associated economic advantages compared to traditional chemical and physical treatment methods [11,38,39].

Bioremediation is defined as ‘the elimination, attenuation or transformation of polluting or contaminating substances by the use of biological processes’ [40]. The applications of bioremediation technologies are commonly targeted at pollutants such as heavy metals, BTEX hydrocarbons, petroleum, polycyclic aromatic hydrocarbons, microaromatics, polychlorinated biphenyls, chlorinated phenols and aliphatics [11,40].

Biofilm-mediated bioremediation has been found to be a more effective and safer alternative to bioremediation with planktonic bacteria since cells growing within a biofilm have higher chances of adaptation to different environments and their subsequent survival [1,3,11]. Biofilms maintain optimal pH conditions, localized solute concentrations and redox potential, allowing cells to improve mineralization processes [11]. Biofilm reactors are generally used to treat hydrocarbons, heavy metals and large volumes of dilute aqueous solutions such as industrial and municipal waste water [11,41].

The important role of EPS in the removal of heavy metals from the environment is due to their involvement in flocculation and ability to bind metal ions from solutions [39]. A major group of bacteria commonly found in metal contaminated waters is sulfate reducing bacteria (SRB). They have been shown to be highly efficient in anaerobic degradation of many organic pollutants and in the precipitation of heavy metals from waste water [41]. Other bacteria applied in the biosorption of toxic heavy metals in bioremediation processes include *Enterobacter* and *Pseudomonas* species [39].

Biofilms play a critical role in the colonization of microbes on minerals by mediating their attachment. The dissolution of metal sulfides is thought to occur in the EPS layer and the methond by which this occurs is based on two assumptions. Firstly, it is assumed that the EPS-complexed iron ions are reduced by an electron tunneling effect. Secondly, ferrous ion-glucuronic acid complexes are not stable, and therefore allow the ferrous ions to migrate into the EPS space. If they diffuse towards the outer membrane, they will be re-oxidised by the enzymatic system of the cells, and can therefore re-enter the oxidation/reduction cycle [42]. In addition, Sand and Gehrke found that there was a correlation between the presence of ferric ions within the EPS layer and the extent of bacterial metabolism [42]. Their results suggested that cells with higher amounts of iron and glucuronic acids within their exopolymers displayed higher oxidation activity than those with low amounts of these components. It has also been reported that when there is lack of nutrients in the environment, the production of EPS is increased to promote the hydrophobic interactions for sorption onto solid surfaces. Their attachment to the substrate would allow greater chances for adsorption of organic trace elements [43].

## 6. EPS roles in bioleaching

In the past few decades, the recovery of metals from low-grade mineral ores using microorganisms has gained increasing popularity due to these bioprocesses being the economical and environmentally friendly [38,44]. This process, termed bioleaching, relies on the ability of microbes to oxidize solid compounds, resulting in soluble and extractable elements [38]. Bioleaching is now an established biotechnological technique for the recovery of heavy metals [45].

*A. ferrooxidans* is an acidophilic, obligately chemolithoautotrophic, gram-negative rod that oxidizes ferrous iron for energy generation [46]. It is one of the most commonly used microorganisms in bioleaching [47,48,49,50]. However, despite many studies of biofilms formed by *A. ferrooxidans* in bioleaching processes [42,50,51,52], the exact chemical structure and physicochemical conditions within the EPS remain unclear [42,53,54].

Early commercial applications of bioleaching processes using this bacterium to recover a variety of metals from low-grade ores [55] included copper [56] and uranium extraction [57]. However, although the roles of *A. ferrooxidans* were known, the mine dumps generated were not conducive to bacterial activity. In 1980, commercial bioleaching applications were developed that aided the activity of the microorganisms involved, resulting in a large number of copper heap bioleaching processes and the biooxidation pretreatment of refractory sulfidic gold ores [49]. Presently, *A. ferrooxidans* and other metal solubilising microorganisms are used in industrial leaching processes for the treatment of low-grade ores, which can contain metal concentrations that are below 0.5% (w/w) [47]. *A. ferrooxidans* are able to oxidize different sulfide minerals including arsenopyrite [48], pyrite, chalcopyrite, galena, sphalerite and pentlandite [58] and the main procedures commercially used are heap leaching, dump leaching, in-situ leaching and reactor leaching [48].

In bioleaching processes, the extent of interaction between the bacteria and mineral surfaces is very important. Attachment of the microorganism to the mineral is followed by the oxidation of ferrous to ferric ions and the reduction of sulfur to sulfate ions [59]. The initial attachment of *A. ferrooxidans* to mineral sulfides is non-specific, and driven by chemotactic [60], electrostatic [61] and hydrophobic and hydrophilic interactions [62]. Once cells have attached to the mineral, a monolayered biofilm is developed over the next few days, covering the cells and mineral surface in an EPS layer. The latter mediates the contact between bacterial cells and the sulfidic energy source, and is therefore an essential step in the formation of biofilms and the subsequent bacteria/substrate interactions [50].

## 7. Chemical composition of EPS produced by *A. ferrooxidans*

The EPS produced by *A. ferrooxidans* consists of neutral sugars, predominantly rhammose, frucose and glucose, and lipids [60]. The chemical constituents of the EPS vary depending on the type of substrate upon which the cells are grown. The mode of attachment also differs as a function of substrate, and hence the expression of different EPS genes will result [50]. The majority of research investigating the nature of the EPS produced by *A. ferrooxidans* has taken place in studies involving pyrite, sulfur and ferrous sulfate substrates [42,50].

### 7.1. A. ferrooxidans EPS produced on pyrite

The exopolymers produced by *A. ferrooxidans* grown on pyrite consists of neutral sugars glucose, rhammose, fucose, xylose and mannose, and C_12﹁﹁_-C_20_ saturated fatty acids, as well as some glucuronic acid residues and complexed ferric ions [45]. The glucuronic acid to ferric ion molar ratio was found to be approximately 2:1, causing the EPS to have a net positive charge therefore allowing the bacteria to attach to negatively charged pyrite via electrostatic interaction [42,45,50]. There is no correlation between the amounts of EPS produced with the resulting levels of bacterial metabolic activity. However, a correlation was found between the total ferric ion content in the EPS and the level of bacterial activity. *A. ferrooxidans* strains that exhibited higher levels of activity were found to possess greater concentrations of ferric ions complexed within their EPS. Since the presence of ferric ions is a prerequisite for attachment to pyrite, cells with more iron were found to adhere more rapidly (and in higher numbers) than cells possessing lower iron concentrations [53]. Cells grown on pyrite also produce greater levels of EPS than cells grown on sulfur and ferrous sulfate, and cells lacking in EPS were found not to attach to, or oxidize, pyrite [50,63]. Furthermore, *A. ferrooxidans* containing higher concentrations of complexed ferric ions within their exopolymers showed stronger electrostatic interactions with pyrite than cells with lower concentrations of ferric ions.

### 7.2. A. ferrooxidans EPS produced on sulfur

The EPS produced by *A. ferrooxidans* grown on sulfur were found to contain a higher concentration of lipids, free fatty acids and phosphorous, but a lower concentration of sugars (only glucose and small traces of glucuronic acid) and virtually no complexed ferric ions or other positively charged groups compared to that grown on pyrite [45,52]. The cells grown on this substrate displayed hydrophobic surface properties and did not attach to charged particles, most likely due to the lack of ferric ion complexes in the EPS [45].

### 7.3. A. ferrooxidans EPS produced on ferrous sulfate

The composition of the EPS produced by *A. ferrooxidans* grown on ferrous sulfate was very similar to that resulting from growth on pyrite [45,50,52]. However, a smaller amount of EPS was produced on the ferrous sulfate because of the observation that cells grown using soluble substrates produce only small amounts of EPS compared to cells grown on solid substrates [60].

## 8. Extracellular polysaccharides produced by pathogenic microorganisms

Pathogenic microorganisms associated with biofilms are the focus of intensive research due to their involvement in a large number of chronic infectious diseases [1,5,13]. Biofilm formation is believed play an important role in infection immunity and protection toward antimicrobial agents [5,64]. For example, the gram-positive *Staphylococcus epidermidis* and the gram-negative *P. aeruginosa* are the most prevalent pathogens involved in clinical chronic infections [13,64]. Their growth and proliferation within a biofilm provides protection from antibiotics and provides them a host defense mechanism by slowing down or preventing penetration of different agents through the biofilm [5,13]. Other biofilm-associated pathogenic bacteria include the generas *Escherichia*, *Legionella*, *Staphylococcus*, *Streptococcus* and *Vibrio* [5,13,30].

## 9. Extracellular polysaccharides employed in biotechnology applications

Some of the well studied, commercially used polysaccharides are produced by taxonomically diverse bacteria [34,35,65,66,67,68]. These polysaccharides, together with their biotechnological applications, are given as follows: 

### 9.1. Extracellular polysaccharides produced by gram-positive bacteria, Firmicutes

The phylum *Firmicutes* has been recognised since 1978, as has its further taxonomic rearrangements [69,70,71]. The bacteria from the orders *Lactobacillales*, *Leuconostocaceae* and *Streptococcaceae* are producers of polysaccharides that are employed in a range of different commercial applications [35,72,73].

#### 9.1.1. Dextran

**Figure 1:**
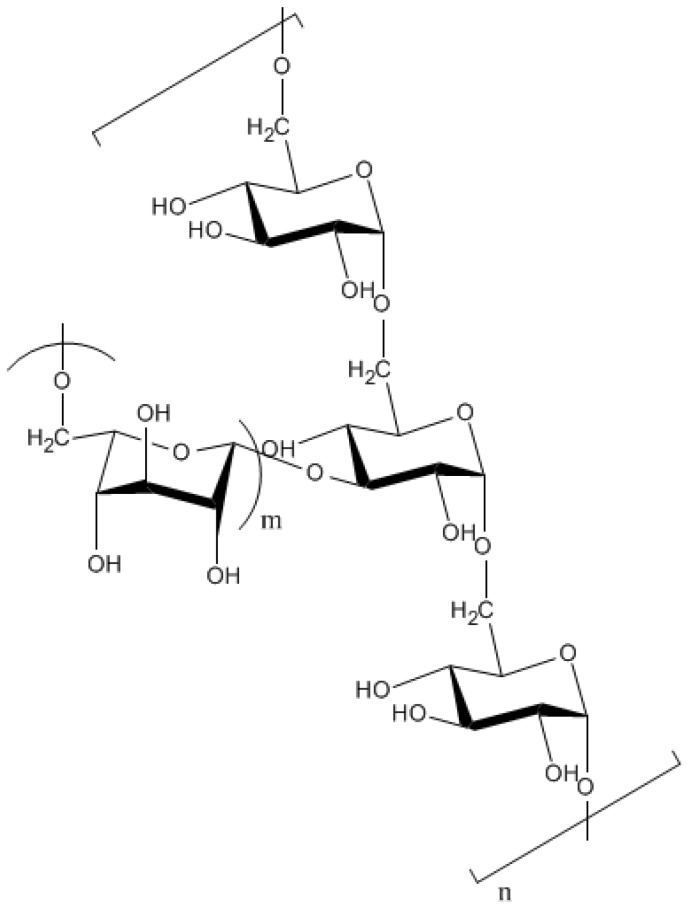
The structure of dextran with branching at C3 [74].

Dextran is a high molecular weight homopolysaccharide of glucose containing numerous consecutive *α-*(1,6)-linkages in its backbone [35]. The *α*-d-glucans have side chains with *α-*(1,6)-linkages, which mainly stem from *α-*(1,3)- and sporadically from *α-*(1,4)- or *α-*(1,2)-linkages (Figure 1). The exact structure of each type of dextran depends on the microbial strain. A majority of dextrans are produced from sucrose by dextransucrase enzymes, synthesized and secreted largely by *Leuconostoc*, *Streptococcus* and *Lactobacillus* species [35,72]. Commercially produced dextran is produced by *L. mesenteroides* and *L. dextranicum­* [35]. The product is a gel that is widely used as a molecular sieve for purification and separation of macromolecules such as proteins, nucleic acids and polysaccharides [72]. Dextran is also used in clinical research and medical applications since it can be safely consumed [35].

#### 9.1.2. Kefiran

**Figure 2:**
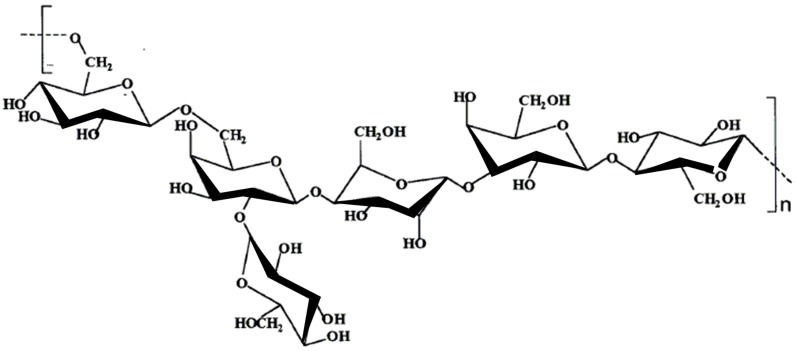
The structure of kefiran, the polysaccharide found in kefir grains [75].

A product that is employed in the dairy industry is kefiran, a capsular polysaccharide produced by *Lactobacillus* species including *L. rhamnosus*, *L. kefir* and *L. kefiranofasciens*, which is found in kefir grains [73,75]. This polysaccharide is considered safe since kefir has been traditionally consumed. Its has also been found to have antibacterial, antifungal and antitumor activity [35,75]. Kefiran is a clear or yellow polysaccharide gel excreted by kefir grains. It is a water-soluble branched glucogalactan with similar ratios of d-glucose and d-galactose residues (Figure 2) [75]. Kefiran is primarily used to produce traditional self-carbonated, slightly alcoholic fermented milk [73] but can also enhance the viscosity and viscoelasticity of acid milk gels [76] and serve to prevent or control come commonly occurring diseases [35].

### 9.2. Extracellular polysaccharides produced by gram-negative bacteria, Proteobacteria

The phylum *Proteobacteria* was established on the basis of phylogenetic analysis of 16S rRNA gene sequences. The phylum contains five classes of gram-negative bacteria including *Alphaproteobacteria*, *Betaproteobacteria* and *Gammaproteobacteria* [77]. The polysaccharides produced by these gram-negative bacteria possess a high degree of structural diversity, consisting of either homopolysaccharides, where polymers are generally composed of d-glucose, or heteropolysaccharides, where the repeating units range from disaccharides to octasaccharides with unusual side chains [68].

#### 9.2.1. Alphaproteobacteria

This class of bacteria are comprised of a heterogeneous group of microorganisms [77]. The main polysaccharides produced include cellulose and gellan, which are primarily produced by bacteria of the orders *Rhodospirillales* and *Sphingomonadales*, respectively.

**Figure 3:**
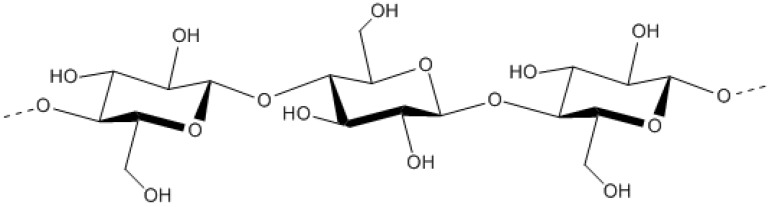
The structure of bacterial cellulose [80].

Cellulose is primarily produced by bacteria of the genera *Acetobacter*, particularly *A. xylinum*, however, bacteria belonging to the *Agrobacterium*, *Pseudomonas* and *Rhizobium* genera can also produce cellulose [78]. Cellulose possesses a simple structure consisting of the monosaccharide glucose in 1→ 4-*β*-glycosidic links (Figure 3) [79]. Bacterial cellulose can be produced as a highly pure polymer, therefore it is used in specific applications to a greater extent than plant cellulose [66]. Cellulose can be utilized in food supplements such as a food matrix, dietary fibre or thickening or suspending agents. The commercially available products Biofill®, Bioprocess® and Gengiflex® are cellulose formulations that are used biomedicine for surgery and wound dressings and dental implants [66,78,80].

**Figure 4:**
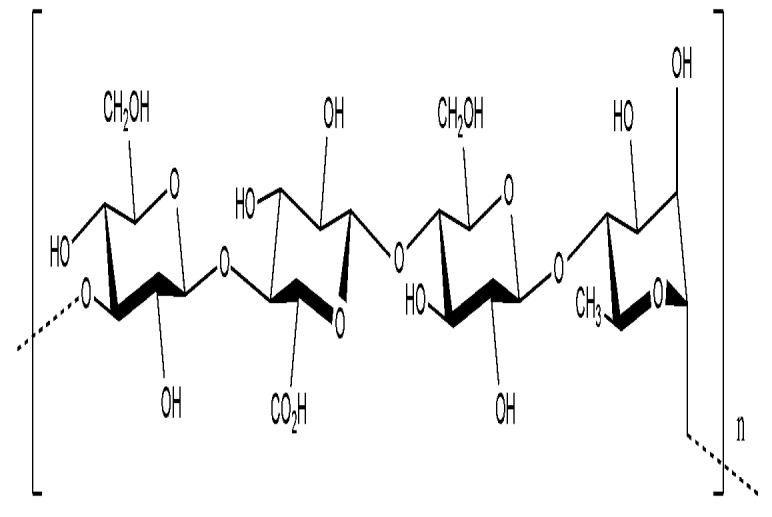
The primary structure of gellan gum [74].

Gellan is a multifunctional gelling agent that is produced by the non-pathogenic bacterium *Sphingomonas elodea* ATCC 31461. It is a linear, anionic polysaccharide consisting of a tetrasaccharide repeating unit comprising of two molecules of d-glucose, d-glucuronic acid and l-rhamnose (Figure 4) [34]. In its native form, gellan forms an elastic gel and in solution, it is able to hold particles in suspension without significantly altering the viscosity of the solution [35]. It also shows thermal and acid stability, elasticity and rigidity, high transparency and good flavour release. Gellan is commercially available as Gelrite, a substitute of agar, and Kelcogel® F and Kelcogel® LT100, which are food-grade gellans. It is also used as an excipient for drug delivery applications [34].

#### 9.2.2. Betaproteobacteria

**Figure 5:**
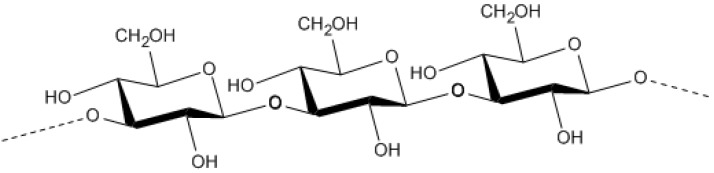
Chemical structure of curdlan [83].

Bacteria of the genus *Alcaligenes*, which belong to the order *Burkholderiales*, are the producers of two commercially available products – curdlan and welan [35]. Curdlan is a low molecular weight, water soluble linear polysaccharide made up of *β*-1,3-linked glucose residues (Figure 5) [35,81]. 

This polysaccharide is produced mainly by *Alcaligenes faecalis* and also *Agrobacterium*. However, commercially produced curdlan comes from a mutant strain of *Alcaligenes faecalis* var. *myxogenes* [35]. Curdlan is unique in its ability to form an elastic gel when its aqueous suspension is heated above 55 ºC [82], making it appealing for use in the food and pharmaceutical industries [81]. It is useful in improving the texture and stability of foods and can be used as a drug delivery polymer [35].

**Figure 6:**
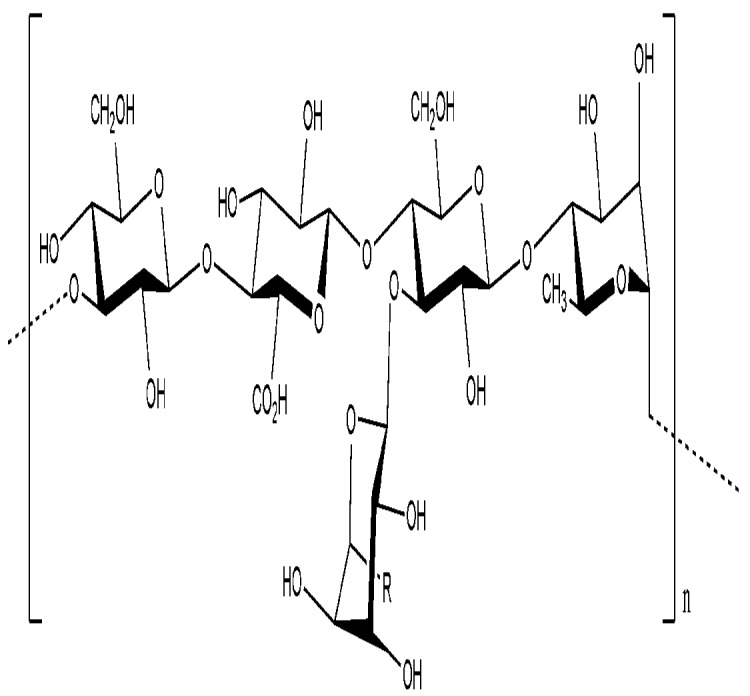
The primary structure of welan gum [74].

Welan is an anionic polysaccharide made up of d-glucose, d-glucuronic acid and l-rhammose units with singular side chains containing either l-rhammose or l-mannose substituted on C3 of every 1,4-linked glucose repeating unit (Figure 6) [35]. It is produced by the *Alcaligenes* species ATCC 31555 and has the same backbone repeating units as gellan gum but with different side chains [74]. Welan retains stability and viscosity at elevated temperatures making it ideal for industrial applications, particularly in oil-well drilling and cement systems as a stabilizer and viscosifier [35,84].

R = CH_3_ or CH_2_OH

#### 9.2.3. Gammaproteobacteria

Commercially available polysaccharides produced by bacteria of this class include bacteria of the orders *Enterobacteriales* (*N*-acetylheparosan), *Pseudomonadales* (alginate) and *Xanthomonadales* (xanthan) [35,77].

**Figure 7:**
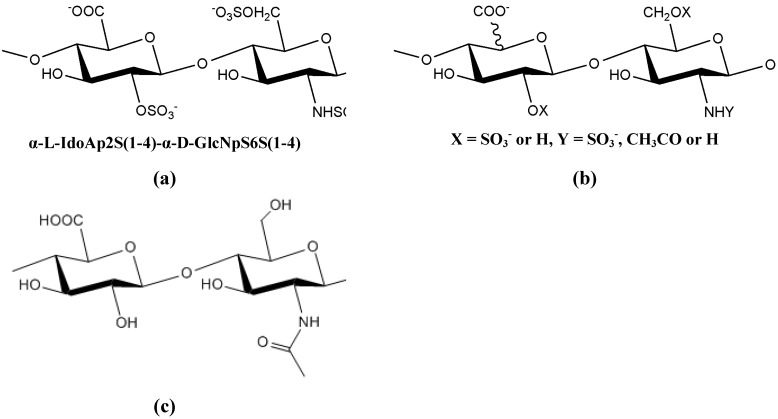
The structure of: (a) the major disaccharide sequence, (b) the minor disaccharide sequence of heparin and (c) the polysaccharide produced by *E. coli* [85].

Heparin is a highly sulfated, linear polysaccharide consisting of alternating d-glucosamine residues 1→ 4 linked to either l-iduronic acid or d-glucuronic acid (Figure 7) [85]. This glycosaminoglycan (GAG) is primarily extracted from porcine intestinal tissues or bovine lung tissue and is widely used as a clinical anticoagulant. However, these mast cells may also nest bacteria or viruses, particularly in bovine heparin, thus limiting the uses of animal heparin [86]. The biosynthesis of heparin requires the formation of non-sulfated polysaccharide chains covalently bound to a protein core, followed by modifications of the polymers [87]. Although microorganisms do not produce heparin [66], the polysaccharide produced by *E. coli* was found to have the same structure, as the non-sulfated precursor polysaccharide in heparin biosynthesis [66,87]. The alternative use of bacterial polysaccharides as sources of heparin synthesis may eliminate concerns associated with mammalian sources of heparin, and also be useful in medical research [85].

**Figure 8:**
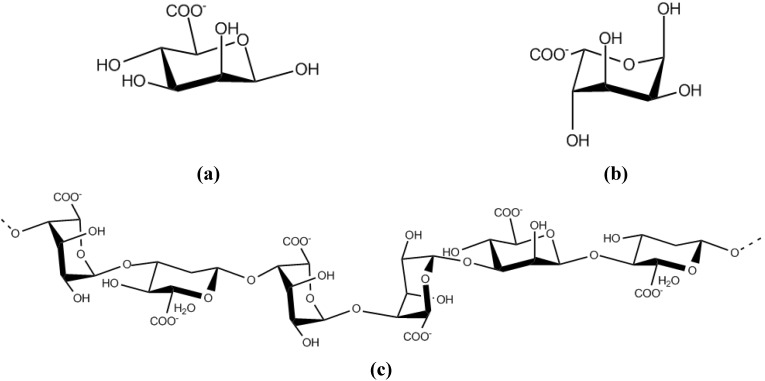
The structure of (a) *β*-d-mannuronic acid, (b) *α*-l-guluronic acid and **(c)** alginate [67].

Alginate is produced by *Pseudomonas* species and *Azotobacter chroococcum* and *A. vinelandii*. It is a linear copolymer comprised of 1-4-linked *β*-d-mannuronic acid (M) and C-5-epimer *α*-l-guluronic acid (G) (Figure 8) [67]. These residues can be arranged in different blocks such as homopolymeric blocks of consecutive G-residues (G-blocks) or M-residues (M-blocks), alternating G and M residues (MG-blocks) or randomly organized blocks. The content of G-blocks stabilizes and improves the gelling characteristics of alginate [35]. Alginate is commercially used in different industrial applications as viscosifiers, stabilizers, gel-forming, film-forming or water-binding agents. It is also used in the pharmaceutical industry as a wound dressings, dental materials and for encapsulation of cells and enzymes for slow release [35,67].

**Figure 9:**
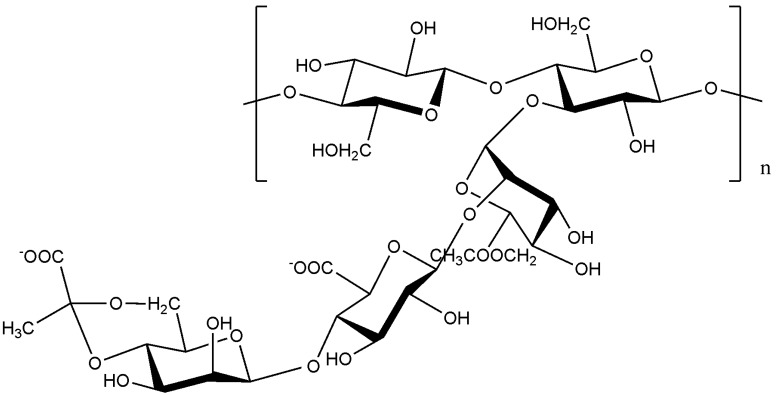
The structure of xanthan [68].

Xanthan is a well studied polysaccharide produced by most strains of *Xanthomonas campestris*. It is a high molecular weight, branched anionic heteropolysaccharide. The main chain is composed of glucose units while the side chain is a trisaccharide, made up of *α﹁*- d-mannose with an acetyl group, *β*-d-glucuronic acid and a terminal *β*-d-mannose unit linked to a pyruvate group (Figure 9) [35]. The trisaccharide side chains align with the backbone via non-covalent interactions to stabilize the structure [66]. Due to its highly pseudoplastic and suspending properties, xanthan is commonly used as a suspending agent and an emulsion stabilizer, primarily in food applications but is also used in cosmetic, pharmaceutical and industrial formulations [35]. 

## 10. Novel techniques in the study of biofilm-specific polysaccharides

A detailed exploration of biofilm matrices, including studies of their thickness, growth and detachment, EPS chemical composition and distribution remains limited due to the lack of sufficiently sensitive analytical techniques [88]. Recent progress in the surface and structure analysis of biofilms has resulted from the development of advanced microscopy and spectroscopy techniques, atomic force microscopy (AFM), confocal laser scanning microscopy (CLSM), infrared spectroscopy, nuclear magnetic resonance imaging (NMRI), Raman spectroscopy (RM) and scanning electron microscopy (SEM) [89,90]. However, combinations of standard methods and the development of novel techniques to study and characterize different biofilms would be useful in many biomedical and industrial research applications [89,91,92].

CLSM is a standard tool used for biofilm analysis. This is a well understood technique, which provides information on the 3D structure of biofilms and aids in the identification and distribution of the different components using fluorescent stains [89]. However, along with the prerequisite of staining the sample, which can have low specificity, CLSM has spatial resolution limitations, thus analyzing the distribution of nanometer-sized biopolymers within the EPS matrix is not possible [93]. When used in conjunction with RM, which gives a deeper insight into the composition and structural information of EPS [94], the requirement for staining is avoided and information on the EPS components can be acquired *in situ* in a non-destructive way. EPS constituents such as polysaccharides and proteins can be chemically classified, and changes in the chemical composition of the aging biofilm matrix, not detectable by CLSM, can be revealed [88].

Although CLSM is the typical method used for investigation of biofilms, it it a technique that only gives information on the distribution and amount of stainable EPS components [88]. On the other hand, AFM is a powerful technique that is capable of imaging the surface morphology under hydrated conditions and in aqueous solutions [94]. This technique requires minimal sample preparation and creates 3D images with nanometer or sub-nanometer resolutions [95]. Since this technique does not require samples to be coated or stained, imaging can be carried out on surfaces in their native state and under physiological conditions. The use of AFM allows visualization of the formation of biofilms and EPS distribution and cells within it [94]. The combination of CLSM and AFM provides an enhanced insight into biofilms with high-resolution images, whereby CLSM can be used for fluorescence imaging and AFM can give a more detailed image of selected sections of the sample [93].

Recently, there has been increasing interest in the application of AFM and RM spectroscopy on the formation of biofilms [94]. The combination of these two powerful techniques allows high resolution visualization of the biofilm and analysis of its components [89]. This allows an analysis of the biofilms and determination of the presence of macromolecules at different stages growth, and hence can provide valuable information regarding bacterial attachment to different surfaces [94].

Optical coherence tomography (OCT) is an emerging, high-resolution medical and biological imaging technology that is able to achieve optical ranging of biological and non-biological structures, similar to that obtained using ultrasound analysis. Since OCT uses short wavelengths of near-infrared light rather than sound, the resolution of the image can be up to 100 times higher than that of standard imaging techniques [96]. OCT involves a depth-resolved analysis of backscattered light using an interferometer that can generate a 2D or 3D image. When this technology is applied to study a biofilm structure, a 3D image of the biofilm developed is reconstructed and a more detailed visualization of structure within the biofilm can be acquired through 2D sections of 3D images at different planes. Although the data obtained can be similar to data collected using other techniques, additional information can be provided using OCT showing that this technology can be a useful imaging tool for characterization of biofilm formation under different conditions [92,96].

Sum-frequency-generation (SFG) spectroscopy is a surface-specific non-linear optical technique used as a complementary technique to characterize biological molecules at interfaces [91,97,98]. This technique has attracted much attention due to its high surface specificity and broad applications. SFG has been successfully applied in areas of surface science including chemistry, biology and materials science, for example, interfaces of pure liquids and electrochemical processes [99]. Howell *et al.* used SFG spectroscopy to study the extracellular matrix under a layer of cells attached to a solid [91]. There were no differences found in the SFG spectra between samples with cells and samples without cells and tests carried out with live cells were not visibly affected by irradiation with SFG laser pulses. These results indicated that the SFG spectroscopy is able to probe the layer between cells and a solid substrate thus this technique could potentially be applied to *in vitro* studies of extracellular matrix under live cells.

## 11. Conclusions

Although microorganisms predominantly exist as multi-cellular communities within biofilms in most environments, scientists are still exploring these complex systems in order to understand the complexity of the interactions within the biofilms, and also their function in bacterial attachment and proliferation. Intense research of many different polysaccharides produced by a diverse range of bacteria has been commercially applied in food and biomedical fields. However, little is known about the role of EPS and biofilms in bioleaching applications. In order to improve the understanding of biofilm systems, the development of sensitive analytical techniques is required. Significant advances have been made to reveal new insights into biofilms and their constituents. The expansion of knowledge in relation to molecular mechanisms involved in bacterial-mineral attachment may be relevant in the enhancement of bioleaching timing and efficiency.
